# Supplementary material for: Distinct Subgroups in Gastroparesis Defined by Simultaneous Body Surface Gastric Mapping and Gastric Emptying Breath Testing
Source: Neurogastroenterol Motil. 2025 Jul 28;37(11):e70124. doi: 10.1111/nmo.70124 (PMC12534583; doi:10.1111/nmo.70124)
Supplement: Supplementary file 1 — Table S1. [file NMO-37-e70124-s001.docx]

**Table S1**: Post-hoc comparisons of symptom severity by gastric emptying breath test status among each body surface gastric mapping phenotype

| **Phenotype** | **Symptom** | **GEBT Status** | | **Delayed GEBT (n)** | **Normal GEBT (n)** | **Mean Difference** | **p** | **Adjusted p value** | **Significance** |
| --- | --- | --- | --- | --- | --- | --- | --- | --- | --- |
| High Frequency | Bloating | Delayed | Normal | 4 | 7 | 1.24 | 0.27 | 1 | ns |
| Delayed Meal Response | Bloating | Delayed | Normal | 9 | 12 | 0.989 | 0.34 | 1 | ns |
| Unstable | Bloating | Delayed | Normal | 11 | 28 | 0.639 | 0.531 | 1 | ns |
| Normal | Bloating | Delayed | Normal | 12 | 60 | 1.77 | 0.0994 | 1 | ns |
| High Frequency | Early Satiety | Delayed | Normal | 4 | 7 | 0.86 | 0.414 | 1 | ns |
| Delayed Meal Response | Early Satiety | Delayed | Normal | 9 | 12 | 1.701 | 0.111 | 1 | ns |
| Unstable | Early Satiety | Delayed | Normal | 11 | 28 | -0.083 | 0.935 | 1 | ns |
| Normal | Early Satiety | Delayed | Normal | 12 | 60 | 3.49 | 0.00372 | 0.10416 | ns |
| High Frequency | Excessive Fullness | Delayed | Normal | 4 | 7 | 2.561 | 0.047 | 1 | ns |
| Delayed Meal Response | Excessive Fullness | Delayed | Normal | 9 | 12 | 0.132 | 0.896 | 1 | ns |
| Unstable | Excessive Fullness | Delayed | Normal | 11 | 28 | 0.87 | 0.394 | 1 | ns |
| Normal | Excessive Fullness | Delayed | Normal | 12 | 60 | 2.99 | 0.0105 | 0.294 | ns |
| High Frequency | Heartburn | Delayed | Normal | 4 | 7 | 0.553 | 0.606 | 1 | ns |
| Delayed Meal Response | Heartburn | Delayed | Normal | 9 | 12 | -0.816 | 0.427 | 1 | ns |
| Unstable | Heartburn | Delayed | Normal | 11 | 28 | 0.171 | 0.866 | 1 | ns |
| Normal | Heartburn | Delayed | Normal | 12 | 60 | 1.195 | 0.252 | 1 | ns |
| High Frequency | Nausea | Delayed | Normal | 4 | 7 | 1.74 | 0.169 | 1 | ns |
| Delayed Meal Response | Nausea | Delayed | Normal | 9 | 12 | 0.414 | 0.687 | 1 | ns |
| Unstable | Nausea | Delayed | Normal | 11 | 28 | 0.616 | 0.548 | 1 | ns |
| Normal | Nausea | Delayed | Normal | 12 | 60 | 3.323 | 0.00532 | 0.14896 | ns |
| High Frequency | Stomach Burn | Delayed | Normal | 4 | 7 | 0.039 | 0.97 | 1 | ns |
| Delayed Meal Response | Stomach Burn | Delayed | Normal | 9 | 12 | 0.846 | 0.412 | 1 | ns |
| Unstable | Stomach Burn | Delayed | Normal | 11 | 28 | -0.483 | 0.635 | 1 | ns |
| Normal | Stomach Burn | Delayed | Normal | 12 | 60 | 1.885 | 0.0823 | 1 | ns |
| High Frequency | Upper Gut Pain | Delayed | Normal | 4 | 7 | 1.53 | 0.183 | 1 | ns |
| Delayed Meal Response | Upper Gut Pain | Delayed | Normal | 9 | 12 | -0.129 | 0.899 | 1 | ns |
| Unstable | Upper Gut Pain | Delayed | Normal | 11 | 28 | 0.906 | 0.377 | 1 | ns |
| Normal | Upper Gut Pain | Delayed | Normal | 12 | 60 | 2.348 | 0.0355 | 0.994 | ns |
